# Potential pulmonary toxic effects of Martian dust simulant

**DOI:** 10.1016/j.isci.2025.113259

**Published:** 2025-08-15

**Authors:** Jie Ji, Jasmine R. Petriglieri, Francesco Turci, Shanzina Sompa, Per Gerde, Lars L. Karlsson, Dag Linnarsson, David J. Loftus, G. Kim Prisk, Urs Staufer, Erin M. Tranfield, Wim van Westrenen, Lena Palmberg

**Affiliations:** 1 Unit of Integrative Toxicology, Karolinska Institutet, Stockholm, Sweden; 2 “G. Scansetti” Interdepartmental Center and Department of Earth Sciences, University of Turin, Turin, Italy; 3 “G. Scansetti” Interdepartmental Center and Department of Chemistry, University of Turin, Turin, Italy; 4 NASA Ames Research Center, Moffett Field, CA, USA; 5 University of California, San Diego, La Jolla, CA, USA; 6 Delft University of Technology, Delft, the Netherlands; 7 VIB Center for Inflammation Research, Ghent, Belgium; 8 Vrije Universiteit Amsterdam, Amsterdam, the Netherlands

**Keywords:** Respiratory medicine, Space medicine, Space sciences

## Abstract

Human exploration of the Moon and Mars will inevitably result in exposure to extraterrestrial dust. We investigated the potential pulmonary toxicity of JSC Mars-1(Martian dust simulant, respirable diameter 1.45 μm) using an advanced multicellular lung mucosa model with dust applied apically to human bronchial epithelial cells cultured at the air-liquid interface, with doses 55, 222, and 890 μg/cm^2^, PBS (sham). Compared to the sham, medium and high doses of JSC Mars-1 increased necrosis-related gene HMGB1 expression. The cellular total reactive oxygen species (ROS) levels increased in a dose-dependent manner. The expression of antioxidant-related genes, HMOX-1 and SOD3, increased at all dose levels. Interleukin-6 (IL-6) protein and gene expression increased, and tumor necrosis factor alpha (TNF-α) after high dose. CXCL8 mRNA was elevated at medium and high doses. TLR4 surface and gene expression decreased at medium and high doses. JSC Mars-1 dust increased cytotoxicity, oxidative stress, and immune responses suggesting potential pulmonary toxic effects, providing insight to molecular mechanisms behind potential adverse respiratory effects.

## Highlights

• Using lung mucosa models to reveal the pulmonary toxicity of Martian dust simulant
• Martian dust simulant induces dose-dependent oxidative stress and immune responses
• Findings advance knowledge of extraterrestrial dust hazards in space exploration

## Graphical abstract

**Figure:**
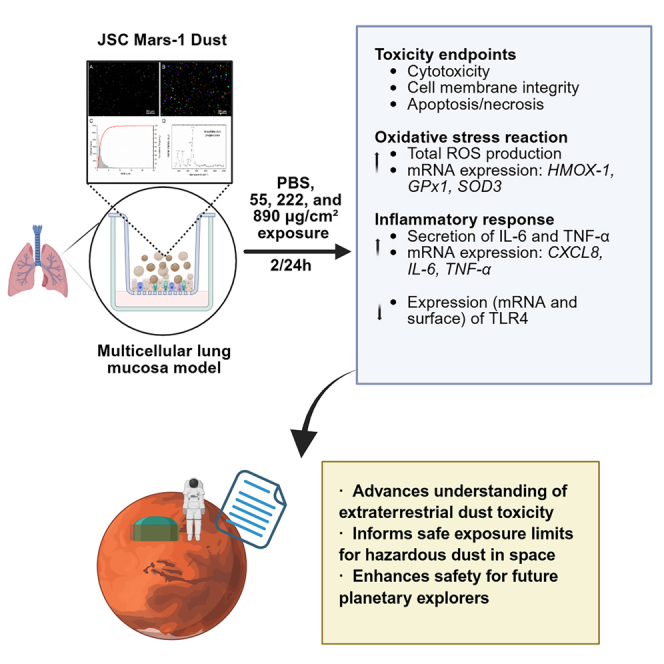

## Introduction

From 1969 to 1972, the crew of six Apollo missions successfully landed on the Moon. In the next few years, NASA, ESA, CNSA, and other space agencies will embark on returning to the Moon as the starting point to reach Mars and even beyond. However, long-duration human space missions and planetary operations pose significant risks associated with exposure to planetary surface dust particles.

A series of distinct environmental factors influence the toxicity of dust on a particular planetary surface.^1^ On Mars, the surface dust is rich in highly oxidized iron and rich in salt.^2^^,^^3^ Perchlorate was also detected at a concentration of approximately 0.5 wt % on Mars soils by the Wet Chemistry Laboratory (WCL) instrument on the Phoenix lander.^4^ The thin atmosphere with its occasional windstorms causes significant dust transport across the surface of Mars. According to the Mars global surveyor thermal emission spectrometer (MGS-TES), the particle size of Mars’s bright surface regions is between 2 and 40 μm, and the dark regions are dominated by particulate materials ranging from 0.1 mm to 1 cm in size.^5^^,^^6^ The commonly accepted value for the effective radius of Martian dust is approximately 1.5 μm.^6^ The smaller the particle, the more easily it can be deposited deep into the human lung. Thus, the high abundance of small particles in Martian dust suggests that a significant portion of the dust can evade the clearance mechanism of the upper and large airways and enter the lower respiratory region and alveoli.^7^ Due to the lower Martian gravity (about 38% of the gravity on Earth), the fraction of the particles remaining in suspension are increased, allowing more particles to reach more peripheral regions of the lung.^8^ Although humans have not yet visited the Martian surface, the unique Martian conditions such as small particle size, reduced gravity, specific surface, and atmosphere properties, are expected to cause inefficient clearance and prolonged residence time of dust particles deposited in the lung, serving to increase the inhalational toxicological effects of Martian dust compared to terrestrial dust.

The ideal material for pulmonary toxicity studies is authentic Martian surface dust, but to date no Mars soil or dust samples have ever been returned to Earth.^9^ Therefore, Martian dust simulants, which are readily available in sufficient quantities, have been widely used for various research purposes. The JSC Mars-1 soil simulant was developed at NASA’s Johnson Space Center in 1998.^3^ It was derived from surficial altered ash of the Pu’u Nene cinder cone, Mauna Kea, in Hawaii.^3^ The particle size of JSC Mars-1 dust was less than 1 mm, and the chemical composition of JSC Mars-1 dust was comparable to that of Martian soil, as both are rich in SO_2_, Fe_2_O_3_, and CaO.^3^ According to the reflectance spectral data obtained by Viking I and II and Pathfinder Landers, the mineralogical properties of JSC Mars-1 dust are analogous to those of the bright regions of Mars, including the presence of glassy basaltic volcanic material and silicate minerals such as plagioclase feldspar.^10^

Animal models have been the primary tool for assessing the inhalation toxicology of planetary dust, such as lunar dust. The Lunar Airborne Dust Toxicity Assessment Group (LADTAG)^11^^,^^12^ reported the toxicity of lunar dust via increased inflammatory biomarkers in bronchoalveolar lavage (BAL) fluid collected from the lower respiratory tract and histopathology changes in the lungs and lymph nodes after nose-only exposure of rats to aerosolized ground lunar material collected during the Apollo 14 mission. Lam et al.^13^ further demonstrated that prolonged exposure to lunar dust simulants led to chronic inflammation in the mouse respiratory system and revealed that the lung toxicity of the lunar dust simulant was more severe than that of titanium dioxide particles. Sun et al.^14^^,^^15^ showed that Chinese lunar dust simulants can induce lung injury and cardiac dysfunction in rats. In comparison, Lam et al.^13^ found that Martian dust simulant causes acute lung toxicity in mice, similar to quartz particles. Although several *in vitro* studies^10^^,^^16^^,^^17^ have reported the cellular toxicity of Martian dust simulants in human keratinocytes, fibroblasts, and macrophages, no study has specifically focused on the pulmonary toxicity of Martian dust on airway epithelial cells. The airway epithelium plays a protective role against inhaled xenobiotics. In our previous studies,^18^^,^^19^^,^^20^ we successfully established physiologically relevant, *in vivo*-like lung mucosa models using primary bronchial epithelial cells cultured at the air-liquid interface. These microphysiological systems offer a unique opportunity for the direct deposition of particles onto a semi-dry apical cell surface, effectively mimicking the cell types found in the lung. In this study, we exposed advanced multicellular lung mucosa models cultured at an air-liquid interface to Martian dust simulant (JSC Mars-1) to investigate the properties of Martian dust from a toxicological perspective and to determine potential health risks.

## Results

**Figure 1:**
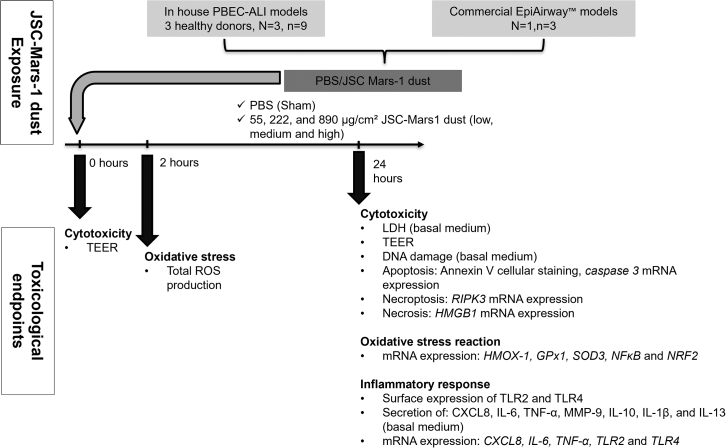
Schematic presentation of the overall experimental design outlining the exposure regimen and endpoints ALI: air-liquid interface; PBEC: human primary bronchial epithelial cells; LDH: lactate dehydrogenase; TEER: transepithelial electrical resistance; RIPK: receptor-interacting protein kinase; HMGB: necrosis marker high-mobility group box; IL: interleukin; TLR: Toll-like receptors; TNF: tumor necrosis factor alpha; MMP: matrix metallopeptidase; ROS: reactive oxygen species; HMOX: heme oxygenase; GPx: glutathione peroxidase; SOD: superoxide dismutase; NF-kB: nuclear factor kappa-light-chain-enhancer of activated B cells; NRF: nuclear factor erythroid 2-related factor.

Figure 1 provides an overview of the experimental design.

### Characterization of JSC Mars-1 dust

#### Particle size distribution analysis

**Figure 2:**
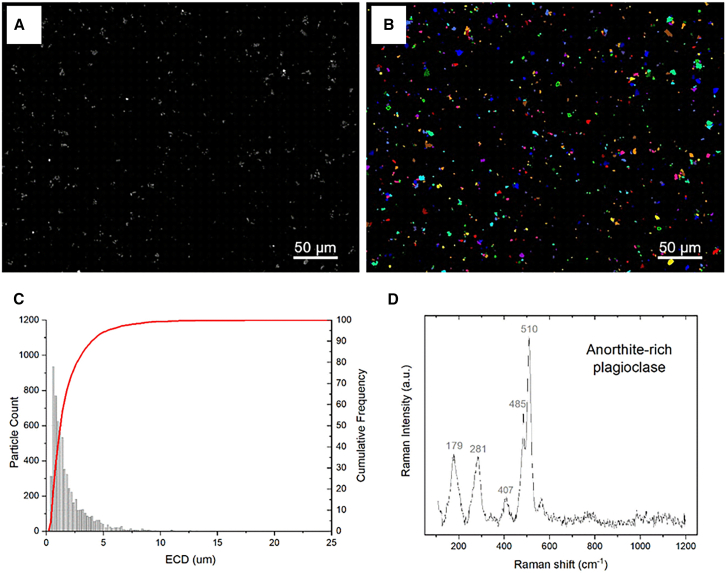
Physico-chemical characterization of JSC Mars-1 dust Representative SEM image (A) and particle classification performed with automatic particle analysis suite (B) of JSC Mars-1 dust. Log-normal type of particle size distribution of JSC Mars-1 dust evidence that 90% of the particle equivalent circular diameters (ECD) are smaller than 5 μm, 50% are smaller than 2.5 μm, and 10% are smaller than 0.5 μm (C). Representative Raman spectrum of anorthite-rich plagioclase particle in JSC Mars-1 dust (D).

Automated image analysis coupled with scanning electron microscopy (SEM) and energy dispersive X-ray spectroscopy (EDS) was performed to characterize the morphometric parameters of the JSC Mars-1 dust after milling, such as the particle shape and size (Figures 2A and 2B). Figure 2C shows the log-normal type of JSC Mars-1 dust particle size distribution. Approximately 90% of the particles were smaller than 5 μm, 50% of the particles were smaller than 2.5 μm, and 10% were smaller than 0.5 μm, considering the equivalent circular diameter (ECD). The median diameter (MD) of the JSC Mars-1 dust was 1.45 μm.

#### Mineralogical identification

The micro-Raman analysis confirmed the mineralogical description reported in the literature,^21^ consisting of basic volcanic glass and plagioclase. A representative Raman spectrum of anorthite-rich plagioclase particles in JSC Mars-1 dust is shown in Figure 2D. The point chemical analyses by EDS (data not reported) were consistent with the phases identified by micro-Raman spectroscopy.

### Cytotoxicity

**Figure 3:**
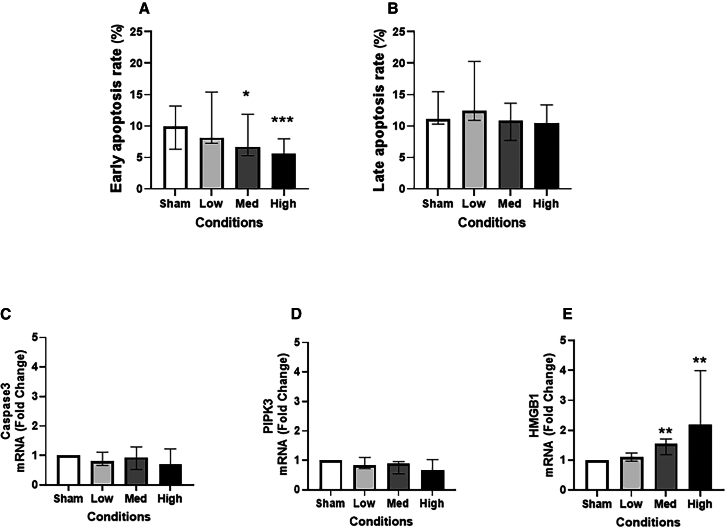
Levels of apoptosis, necroptosis, and necrosis markers post JSC Mars-1 dust exposure Cellular early (A) and late (B) apoptosis rate detected by FACS after exposure to JSC Mars-1 dust and incubated for 24 h (*N* = 4, *n* = 12); The fold change of mRNA expression of apoptosis marker: caspase-3 (C), necroptosis marker: receptor-interacting protein kinase 3 (RIPK3) (D), necrosis marker: high-mobility group box 1 (HMGB1) (E) after exposed to JSC Mars-1 and incubated for 24 h (*N* = 4, *n* = 8); Data presented as median and 25^th^−75^th^ percentiles; Fold change = 2-ΔΔCt; ∗,∗∗,∗∗∗: *p* < 0.05, 0.01, 0.001 VS Sham exposure; Med: medium.

According to the fluorescence-activated cell sorting (FACS) data obtained 24 h post-exposure, the models exposed to high and medium doses of JSC Mars-1 dust had lower rates of early apoptosis (Figure 3A) than the sham exposure models. However, there was no such difference between the sham and exposure groups regarding the late apoptosis rate (Figure 3B), the mRNA expression of the apoptosis-related gene *caspase 3* (Figure 3C), or the necroptosis-related gene *RIPK3* (Figure 3D). The gene expression level of the necrosis marker *HMGB1* (Figure 3E) increased in a dose-dependent manner after exposure to different concentrations of JSC Mars-1 dust, with a significant increase in the medium- and high-exposure groups compared with the sham group (by 2-fold and 3.8-fold [both *p* < 0.01]).

Lactate dehydrogenase (LDH) analysis and transepithelial electrical resistance (TEER) testing revealed no changes in cell viability with or without exposure to JSC Mars-1 dust (Figures S1A and S1B). To investigate DNA damage, we measured 8-hydroxy-2-deoxyguanosine levels in cell culture supernatants, and exposure to different concentrations of JSC Mars-1 dust did not induce DNA damage (Figure S1C).

### Oxidative stress response

**Figure 4:**
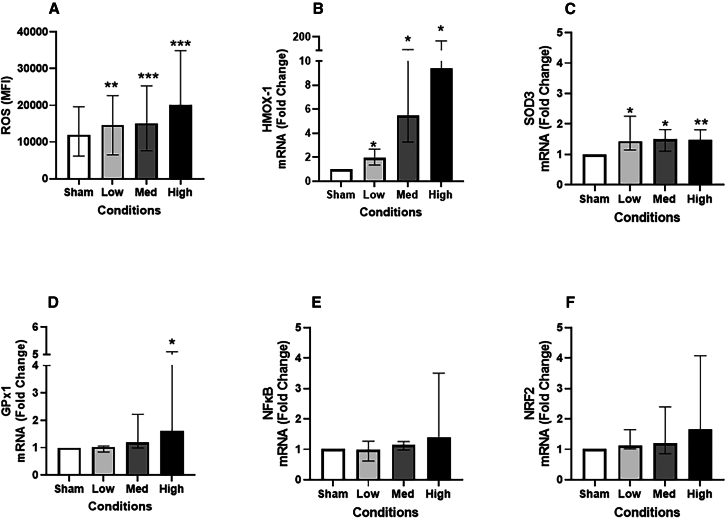
JSC Mars-1 dust exposure induced both the ROS production and mRNA expression of oxidative stress related genes The production of ROS (A) after exposure to JSC Mars-1 dust and incubated for 2 h (*N* = 4, *n* = 12); The fold change of mRNA expression of oxidative stress related gene HMOX1 (B), SOD3(C) GPx1 (D), NF-κB (E), and NRF2 (F) after exposure to JSC Mars-1 dust and incubated for 24 h (*N* = 4, *n* = 8); Data presented as median and 25^th^−75^th^ percentiles; Fold change = 2-ΔΔCt; ∗, ∗∗,∗∗∗: *p* < 0.05, 0.01, 0.001 VS Sham exposure; Med: medium.

The cellular production of ROS (Figure 4A) was higher 2 h after exposure to all three doses of the JSC Mars-1 dust, than after sham exposure, and ROS production strongly increased in a dose-dependent manner with fold changes of 1.2-fold (*p* < 0.01), 1.3-fold, and 1.7-fold (*p* < 0.001). In terms of the antioxidant response to JSC Mars-1 dust, the mRNA expression of *HMOX-1* (Figure 4B) increased with fold changes of 2.9-fold, 11.8-fold, and 85.9-fold (all *p* < 0.05), after exposure to all tested concentrations of JSC Mars-1 dust. Similarly, the mRNA expression of *SOD3* (Figure 4C) was increased with fold changes of 2.3-fold, 1.9-fold, (both *p* < 0.05), and 2.2-fold, (*p* < 0.01) after low, medium, and high dose of JSC Mars-1 dust exposure. The *GPx1* gene expression (Figure 4D) increased significantly (*p* < 0.05) upon exposure to the highest concentration of JSC Mars-1 dust.

The expression of major transcription factors related to maintaining redox homeostasis in cells, such as *NF-κB* (Figure 4E) and *NRF2* (Figure 4F), did not significantly differ between the different exposure groups, but tended to increase with high-dose exposure.

### Inflammatory reaction

**Figure 5:**
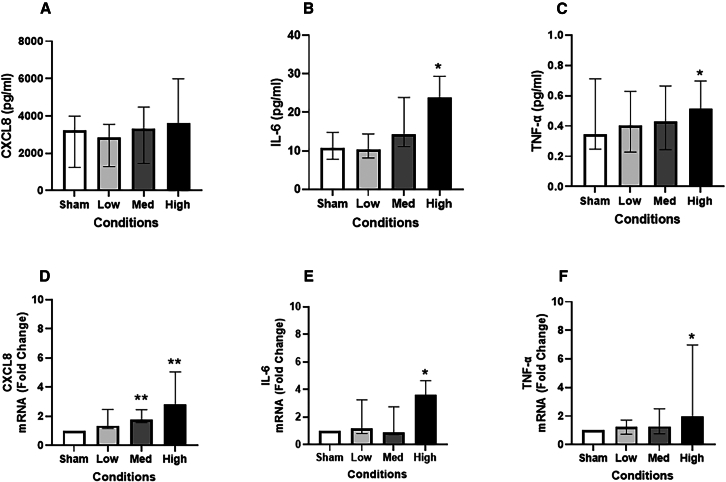
JSC Mars-1 dust exposure induced both release and mRNA expression of inflammatory cytokines Levels of CXCL-8 (A), IL-6 (B), and TNF-α (C) secretion in basal medium (*N* = 4, *n* = 12) and the fold change of mRNA expression of CXCL-8 (D), IL-6 (E), and TNF-α (F) (*N* = 4, *n* = 8) after exposed to JSC Mars-1 dust and incubated for 24 h; Data presented as median and 25th −75th percentiles; Fold change = 2-ΔΔCt; ∗, ∗∗: *p* < 0.05, 0.01 VS Sham exposure; Med: medium.

Both the protein and gene expression levels of IL-6 (Figures 5B and 5E) (2-fold at protein level, 3.9-fold at gene level) and TNF-α (Figures 5C and 5F) (1.4-fold at protein level, 8.2-fold at gene level) were significantly (*p* < 0.05) increased after exposure to the highest dose of JSC Mars-1 dust compared to those in the sham group. *CXCL8* mRNA expression was significantly greater in both medium and high-exposed groups than in the sham-exposed group, with fold changes of 2-fold and 3.4-fold (*p* < 0.01) (Figure 5D). MMP9 and IL-10 secretion were not significantly affected (data not shown), whereas IL-13 and IL-1β concentrations were below the detection limits in most samples.

**Figure 6:**
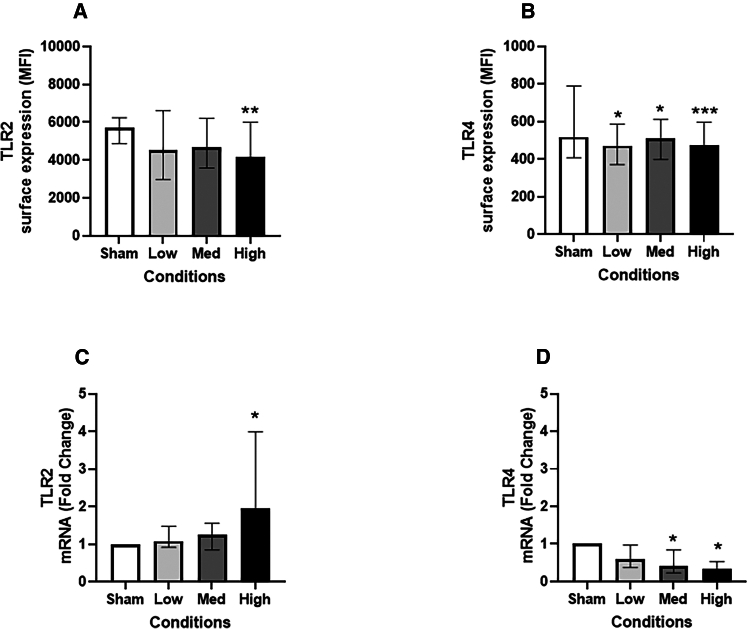
Surface and mRNA expression of Toll-like receptors post JSC Mars-1 dust exposure Surface expression of TLR2 (A) and TLR4 (B) (*N* = 4, *n* = 12); The fold change of TLR2 (C) and TLR4 (D) mRNA expression (*N* = 4, *n* = 8) after exposed to JSC Mars-1 dust and incubated for 24 h; MFI: mean fluorescence intensity; Data presented as median and 25^th^−75^th^ percentiles; Fold change = 2-ΔΔCt; ∗: *p* < 0.05 VS Sham exposure; Med: medium.

The surface expression of toll-like receptor (TLR) 2 (Figure 6A) was significantly (*p* < 0.01) reduced at the highest JSC Mars-1 dust exposure dose. However, the mRNA expression of *TLR2* (Figure 6C) tended to increase in a dose-dependent manner, with a significant (*p* < 0.05) increase in the high-exposure group compared with the sham group. TLR4 cell surface expression (Figure 6B) decreased with fold changes of 1.1-fold (*p* < 0.05) and 1.2-fold (*p* < 0.001), and *TLR4* gene expression (Figure 6D) also decreased with fold changes of 2-fold and 2.8-fold (both *p* < 0.05) in the medium- and high-dose JSC Mars-1 treated groups compared to the sham group.

## Discussion

Since the first debriefings of Apollo astronauts, the potential toxicity of planetary dust exposure has been one of the major health concerns in human space exploration. Our study demonstrates that exposure to JSC Mars-1 triggers different cellular responses in advanced lung mucosa models, including cytotoxicity, oxidative stress, and inflammation. By employing physiologically relevant *in vitro* systems, our work facilitates a more accurate evaluation of pulmonary effects induced by planetary dust and establishes a valuable platform for future research into space-related health risks.

The dose range of JSC Mars-1 dust (55–890 μg/cm^2^) used in this study was determined based on cytotoxicity testing conducted in 2D experiments. No reduction in cell viability was detected by LDH testing, nor did we observe striking DNA damage. TEER measurements also confirmed integrity of the cell barrier after exposure.^22^ These findings support the selection of this dose range to maximize biological effects while minimizing cytotoxicity, providing a relevant framework for evaluating cellular responses to Martian dust simulant under controlled conditions. Although several studies^11^^,^^12^^,^^23^ have established a preliminary permissible exposure limit of 0.3 mg/m^3^ for lunar dust based on safe exposure estimates and the relative toxicities of TiO_2_ and quartz, accurately determining exposure levels in extraterrestrial environments remains challenging due to factors, such as particle size distribution, resuspension dynamics, and individual astronaut activities. Our results are also in accordance with those of an earlier study^16^ in which no cytotoxic effects were observed for Martian dust simulant suspensions applied to HaCaT keratinocytes at the similar exposure concentration ranges. However, in contrast to the findings of Wise et al.,^17^ where the Martian dust simulant (Mars-1A) reduced the relative survival rate of human skin fibroblasts by more than 50% at a concentration of 100 μg/cm^2^, our study showed lower toxicity outcomes. This difference may be attributed to the variation in particle size. In Wise et al.’s study, the mass median diameter (MMD) of the Mars-1A was 0.133 μm, which is smaller than the MMD of the JSC Mars-1 dust (1.45 μm) used in our present work. The smaller particle size observed in Wise et al.’s study corresponds to a very high surface-to-volume ratio, which might not accurately represent the particle sizes encountered in actual planetary environments. In contrast, the particle size used in our study, with an MMD of 1.45 μm, more closely aligns with the range of particle sizes likely to be encountered on Mars.^24^

In our study, after 24 h the early cellular apoptosis rate was lower in the medium and high JSC Mars-1 dust exposure groups than in the sham group. However, there were no changes in late cellular apoptosis, the expression of an apoptosis-related gene (casepase 3) or the expression of a gene that is a critical regulator of programmed necroptosis (RIPK3). These results are somewhat in contrast to our expectations. According to Latch et al.,^10^ Martian dust simulant concentrations ranging from 100 to 500 μg/mL induced a concentration-dependent increase in apoptosis. However, similar to our results, one study^25^ reported a non-monotonic manner of apoptosis after treating A549 cells with different concentrations of montmorillonite particles. They concluded that, instead of apoptosis, necrosis is the dominant death mechanism at higher concentrations. We speculate that this might also be the reason why we observed a decrease in the early apoptosis rate after medium and high JSC Mars-1 dust exposures. This hypothesis is supported by our results regarding the mRNA expression of *HMGB1*, which is normally released passively by necrotic cells. The gene expression of *HMGB1* gradually increased as the JSC Mars-1 dust dose increased, with significant upregulation after medium- and high-dose exposures. Furthermore, apoptotic cells generally tend to be immunologically silent and may not trigger inflammation, while necrotic cells can provoke inflammatory reactions by secreting different cytokines.^26^^,^^27^ The decrease in apoptosis and increase in necrosis caused by the JSC Mars-1 dust were also consistent with the increase in the gene and protein levels of TNF-α after exposure to the high JSC Mars-1 dust in the present study. It is important to note that this study assessed only a limited number of necrosis-related markers. Future research incorporating more comprehensive necrosis-related gene panels and protein-level validation approaches, such as immunostaining, is warranted to further elucidate the mechanisms of cell death.

It is generally believed that when in contact with biological fluids, planetary dust can generate free radicals, including reactive oxygen species (ROS).^28^^,^^29^ An imbalance between ROS production and antioxidant defense leads to oxidative stress, a key mechanism underlying the toxicity of lunar dust particles. Kaur et al.^29^ demonstrated a sustained generation of ROS when a lunar simulant was dispersed in simulated lung fluid. Their findings further indicate that exposure to these particles reduces lung epithelial cell viability and induces DNA damage, likely driven by ROS-mediated oxidative stress. Another study also has shown that lunar dust can activate and upregulate nicotinamide-adenine dinucleotide phosphate oxidases 4 (NOX4) and 2 (NOX2), contributing to increased ROS generation^30^ Therefore, in the present study, we detected total cellular ROS, 2 h after JSC Mars-1 dust exposure and detected a significant (*p* < 0.01 for low and medium dose and *p* < 0.001 for high dose) dose-dependent increase. In addition, we detected a dramatic upregulation of antioxidant gene expressions after JSC Mars-1 dust exposure compared with that in the sham group (*HMX O-1* and *SOD3* after all three dose exposures and *GPx1* after high exposure). In particular, the level of *HMOX-1*, which was significantly affected by the JSC Mars-1 dust, increased by more than 10-fold in the high-exposure group compared with the sham group. In line with our study, an earlier study^31^ demonstrated that a suspension of lunar regolith simulant at 1.0 mg/mL can induce both ROS production and *HMOX-1* gene expression in A459 after 24 h of culture. Recently, Harrington et al.^32^ defined an indicator (the inflammatory stress response) to standardize ROS production in cells after exposure to different dusts. Using this technique, they evaluated the relative toxicity of meteorite samples (Moon, Mars, and Asteroid 4Vesta) and reported that the highest inflammatory stress response and cellular upregulation of ROS were generated by the Mars regolith.^33^ Similar findings have been shown in animal experiments, where the number of oxidants per bronchoalveolar lavage fluid neutrophil in rats increased in a dose/time-dependent manner after Apollo-14 lunar soil intratracheal instillation.^34^ Additionally, in male Wistar rats ROS (increased malondialdehyde) were produced in lung tissue 4 and 24 h after a single exposure to a lunar soil simulator.^14^ All the aforementioned results indicate the development of oxidative stress in the lung after exposure to Martian dust, which may damage cells and tissues, leading to pulmonary toxicity. However, our current study focuses solely on acute exposure at a single time point. To fully understand the progression and long-term impact of oxidative damage, future studies incorporating chronic exposure scenarios and comprehensive time course analyses are warranted.

Extensive research over the past two decades has identified a significant “immune challenge” associated with spaceflight, characterized by immune dysregulation. Spaceflight studies have indicated that exposure to lunar dust can disrupt immune homeostasis by suppressing T cell activation, shifting leukocyte distribution, and alternating cytokine profiles.^35^ This dysregulation can lead to an exaggerated inflammatory response, with biomarkers such as CXCL8 and TNF-α that play key roles in provoking inflammation. Additionally IL-6 is a pro-inflammatory cytokine associated with both local and systemic inflammation that may lead to different lung diseases.^36^ Our results demonstrated the induction of inflammatory gene expression and protein secretion following 24 h of exposure to JSC Mars-1 dust in our advanced lung mucosa models. This induction was evidenced by the increased secretion of IL-6 and TNF-α, accompanied by elevated mRNA expression levels of *CXCL8*, *IL-6*, and *TNF-α*. Consistent with our results, one study^31^ applied a lunar regolith simulant to A549 cells at concentrations of 0.1 and 1.0 mg/mL and reported the induction of CXCL8, IL-1β, and TNF-α secretion after 24 h of exposure. However, Li et al.^37^ reported that lunar soil simulant (JSC-1A) exposure decreased pro-inflammatory gene expression but induced an anti-inflammatory phenotype in macrophages. In addition, Latch et al.^10^ demonstrated that both lunar simulant JSC-1 and JSC Mars-1 can change macrophage subpopulations transitioning from an immune-active state to a suppressive state, which exerts anti-inflammatory effects. The difference in inflammatory reactions between epithelial cells and macrophages may be due to the overload of macrophages with particles that they are unable to clear, which may impair the inflammatory response. Additionally, animal studies have indicated that intratracheal administration of planetary dust simulant can cause damage to the lung epithelium and the development of both local and systemic inflammatory responses. One study where intratracheally administered JSC-Mars1 to C57BL/6J mice reported an increased percentage of neutrophils in BAL fluid.^13^ Acute intratracheal instillation of a lunar soil simulator in male Wistar rats also caused a significant increase in TNF-α and IL-6 in the serum and BAL fluid.^14^^,^^15^ In addition, a recent study^38^ reported dose- and time-dependent changes in inflammation-associated gene expression in lung tissues of Fisher 344 male rats exposed to lunar dust. Taken together, these findings indicate that JSC-Mars1 can induce cytokine and chemokine responses at both the expression and secretion levels, and the upregulation of inflammatory biomarkers suggests a potential for immune dysregulation. This highlights the risks associated with extraterrestrial dust exposure, which may contribute to lung damage, disease development, and weakened immune defenses during space missions.

TLR2 and TLR4 play critical roles in regulating innate and adaptive immunity during particle exposure.^39^ In our current study, we found that when cells were exposed to high levels of the JSC Mars-1 dust, the surface expression of TLR2 on the bronchial epithelial cells decreased, while its mRNA expression increased. Both the cell surface expression and mRNA expression of TLR4 were reduced in the JSC Mars-1 dust-exposed groups, except for the mRNA expression in the low-exposure group. These results are partly in line with our previous study,^19^ in which TLR2 mRNA expression increased while TLR4 surface expression decreased in our bronchial lung mucosa models after diesel exhaust particle exposure. In contrast, one study^40^ reported that *TLR2* mRNA levels were not altered, while *TLR4* mRNA expression was significantly increased in normal human bronchial epithelial cells exposed to particulate matter (PM). A possible explanation for this difference might be the different cell culture methods, since in their study, submerged cell culture was used, and particles were added directly to the cell culture medium, which increases the possibility of particle agglomeration and may lead to unreliable outcomes. Interestingly, in the same study,^40^ with macrophages under the same exposure conditions, TLR2 was upregulated, while TLR4 mRNA expression was downregulated, which was consistent with our findings. Similarly, in myeloid DCs, the cell surface expression of both TLR2 and TLR4 was significantly downregulated after exposure to particulate airborne pollution.^41^ Therefore, the reduction in TLR expression in response to JSC Mars-1 dust may constitute negative feedback to limit increased inflammatory and oxidative stress and avoid subsequent cellular damage. However, to determine the mechanisms underlying the alterations in TLR induced by JSC Mars-1 dust, we believe additional experiments are needed. For instance, specific genes involved in TLR pathways can be silenced.

To our knowledge, this is the first study to reveal the possible pulmonary toxicity of a Martian dust simulant in epithelial cells, particularly using *in vivo*-like mucosal models. We provide description of the cytotoxic effects, oxidative stress reactions, and immune responses in these lung models that were significantly induced by exposure to planetary dust. Our results can advance the understanding of cellular mechanisms that may explain why exposure to planetary dust is harmful to human health. Furthermore, our findings might be useful for setting appropriate dust exposure limits and ultimately helping to ensure the safety of future planetary explorers.

### Limitations of the study

Most *in vitro* studies evaluating the toxicity of planetary dust simulants have relied on submerged monoculture *in vitro* studies,^10^^,^^16^^,^^17^^,^^31^ which fail to capture the complex cell to cell communication and direct dust-cell interactions that occur *in vivo*. In this study, we utilized advanced multicellular ALI models including primary human epithelial cells incorporating diverse cell types, providing a more realistic platform for the direct deposition of Martian dust simulants onto a semidry apical surface, closely simulating the real-life inhalation process. Furthermore, in our experimental setup, both in-house and commercial ALI models were used, and both exhibited consistent result trends, underscoring the robustness of our findings. However, this study has several limitations. Firstly, our investigation is confined to evaluating the effects of Martian dust simulants on lung tissue alone, which represents only one organ of the human body. To gain a more comprehensive understanding of systemic effects, future research should include more complex models that incorporate immune cells and assess the impact of Martian dust simulant on additional organs. Secondly, our experiments primarily focused on short-term inflammatory responses, leaving the potential long-term effects, such as chronic inflammation, immune dysregulation, and fibrotic remodeling, unexplored. As astronauts on extended missions may experience cumulative dust exposure, that could compromise respiratory and systemic immunity, future studies should investigate prolonged exposure scenarios to better understand the potential health risks over time. However, fully replicating chronic exposure conditions on Martian remains challenging due to key environmental differences in space, such as microgravity, radiation, and prolonged confinement, all of which could significantly influence immune responses. Another limitation is lack of perchlorate in the JSC Mars-1 dust. Given that perchlorate can influence thyroid function, it is crucial for future studies to investigate its potential toxicity in detail to fully elucidate the risks associated with Martian dust.

## Resource availability

### Lead contact

Further information and requests for resources and reagents should be directed to and will be fulfilled by the lead contact, Jie Ji (jie.ji@ki.se).

### Materials availability

This study did not generate new unique reagents.

### Data and code availability

• This article does not report any original code.
• Any additional information required to reanalyze the data reported in this paper is available from the lead contact upon request.
• No additional resources are reported.

## Acknowledgments

This study was supported by grants from the 10.13039/501100003793Swedish Heart-Lung Foundation (LP:20220172 and 20210211), the 10.13039/501100004359Swedish Research Council (LP: 2018-03233), and the 10.13039/100009487Swedish Fund for Research Without Animal Experiments (LP: F34-14 and F36/15). This study was also supported by the ESA Topical Team on the Toxicity of Celestial Dust (T3CD).

## Author contributions

J.J., J.R.P., F.T., S.S., P.G., L.L.K., D.L., D.J.L., G.K.P., U.S., E.M.T., W.v.W., and L.P. contributed to the conception; J.J., J.R.P., F.T., P.G., and L.P. designed the work; J.J., J.R.P., F.T., S.S., and L.P., data acquisition and analysis; J.J., J.R.P., F.T., S.S., P.G., L.L.K., D.L., D.J.L., G.K.P., U.S., E.M.T., W.v.W., and L.P. interpreted the data; J.J., J.R.P., F.T., S.S., P.G., L.L.K., D.L., D.J.L., G.K.P., U.S., E.M.T., W.v.W., and L.P. drafted and revised the manuscript;J.J., J.R.P., F.T., S.S., P.G., L.L.K., D.L., D.J.L., G.K.P., U.S., E.M.T., W.v.W., and L.P. approved the submitted version.

## Declaration of interests

P.G. is a minority shareholder in Inhalation Sciences Sweden AB.

## STAR★Methods

### Key resources table

**Table:** 

REAGENT or RESOURCE	SOURCE	IDENTIFIER
**Antibodies**		

Toll-like receptor (TLR)2	BD Biosciences	Cat#742770
Toll-like receptor (TLR)4	BD Biosciences	Cat#564401:RRID:AB_2738792

**Chemicals, peptides, and recombinant proteins**		

Hydrocortisone Stock Solution	Stemcell Technologies	Catalog # 07925
Penicillin‒streptomycin antibiotics (PEST)	Thermo Fisher Scientific	Catalog #15140122
Phosphate-buffered saline (PBS)	Thermo Fisher Scientific	Catalog #10010023
Heparin Solution	Stemcell Technologies	Catalog # 07980
Quartz Min-U-Sil 5	US Silica Company	Catalog# Min-U-Sil 5
Tungsten carbide (WC)	Johnson Matthey	Catalog# 625655
Martians dust simulant (JSC Mars-1)	US NASA	Courtesy Dr. J.D. Loftus

**Critical commercial assays**		

PneumaCult™-Ex expand medium	Stemcell Technologies	Catalog # 05008
PneumaCult™-ALI maintenance medium	Stemcell Technologies	Catalog # 05001
EpiAirway™ culture medium	MatTek	Catalog #AIR-200-M125
Pierce Chromogenic Endotoxin Quant Kit	Thermo Fisher Scientific	Catalog #A39553
Lactate dehydrogenase (LDH) assay kit	Thermo Fisher Scientific	Catalog #C20301
8-hydroxy-2-deoxyguanosine ELISA kit	Thermo Fisher Scientific	Catalog #EEL004
CellROX™ Green Reagent kit	Thermo Fisher Scientific	Catalog #C10444
FITC annexin V apoptosis detection kit II	BD Biosciences	Catalog #556570
Interleukin 8 (IL-8/CXCL8) DouSet ELISA kit	R&D Systems	Catalog #DY208
Matrix metallopeptidase 9 (MMP-9) DouSet ELISA kit	R&D Systems	Catalog #DY911
Meso Scale Discovery (MSD) V-PLEX kit	Mesoscale	Catalog #K15052D
RNeasy Micro Kit	Qiagen	Catalog #74004
cDNA synthesis kit	Applied Biosystems	Catalog #4388950
SYBR Green master mix	Thermo Fisher Scientific	Catalog #4309155

**Experimental models: Cell lines**		

Primary bronchial epithelial cells (PBEC)	In house	N/A
EpiAirway™ model	MatTek	Catalog #AIR-200-PE12

**Oligonucleotides**		

Primers for: caspase 3, RIP3, HMGB1, CXCL-8, IL-6, TNF-α, TLR2, TLR4, HMOX-1, GPx1, SOD3, NFκB and NRF2, see Table S1	This paper	

**Software and algorithms**		

FlowJo software	FlowJo LLC.	N/A
GraphPad Prism software	GraphPad Software, Inc.	N/A
OriginPro 2023 (64-bit)	OriginLab Corporation	10.0.0.154 (Academic)

**Other**		

0.4 μM pore size transwell inserts	BD Falcon™	Catalog #353180
Planetary ball mill	Fritsch	PULVERISETTE 6

### Experimental model and study participant details

#### Multicellular lung cell models

In-house ALI models: Primary bronchial epithelial cells (PBEC) were harvested from bronchial tissues from 3 human donors who underwent lobectomy after providing informed and written consent. The study was performed following the approval of the Swedish Ethical Review Authority (Institutional ethic committee reference number 99-357). No further donor information (such as age, sex, gender, ancestry, or ethnicity) is available to us. The cells used in this study are well characterized and have also been used in other studies.^18^^,^^19^^,^^20^ Multicellular PBEC models were developed as previously described.^18^^,^^19^^,^^20^ Briefly, PBEC were seeded onto 0.4 μM pore size transwell inserts (BD Falcon™) at a density of 1×10^5^ cells/cm^2^. The models were grown under submerged conditions until confluence (95%) in PneumaCult™-Ex expand medium (supplemented with 96 μg/ml hydrocortisone and 1% penicillin‒streptomycin antibiotics (PEST); Stemcell Technologies, Cambridge, UK). Then, the apical medium was removed to achieve airlifting, and the basal medium was replaced with 1 ml of PneumaCult™-ALI maintenance medium (supplemented with 96 μg/ml hydrocortisone, 2 mg/ml heparin and 1% PEST; Stemcell Technologies, Cambridge, UK). The maintenance medium was changed every second day, and phosphate-buffered saline (PBS) was used to rinse the apical side of the model to reduce mucus accumulation. The cell models were airlifted and cultured in 5% CO_2_ at 37°C for 2-3 weeks. Before exposure, the number of PBEC reached ∼1.5x10^6^ cells/insert, and they differentiated into different cell types, including ciliated cells, goblet cells, club cells, and basal cells mimicking the cell types seen in the lung.

EpiAirway™: In our experiment, we also utilized the EpiAirway™ model (AIR-200-PE12) produced by MatTek (Ashland, MA, USA) to complement our primary cell-based in-house ALI model. As these are commercially available tissues, specific donor information is not accessible to us. This commercially available organotypic model of human airway epithelium was instrumental in enhancing the robustness and reliability of our experimental setup. Upon arrival, these ready-to-use cultures were separated from agarose and transferred to 12-well plates with fresh medium (AIR-200-M125; MatTek). Before exposure, these tissue models were equilibrated in 5% CO_2_ at 37°C for 16–18 hours following the manufacturer’s instructions. More information and additional peer-reviewed research articles regarding EpiAirway™ can be found on the MatTek website (www.mattek.com).

In the results section, we present combined data from both our in-house model (individual experiments (N=3), repetitions within each experiment (n=9)) and the EpiAirway™ model (N=1, n=3).

### Method details

Figure 1 provides an overview of the experimental design, including JSC Mars-1 dust (kindly provided by Dr. D.J. Loftus – NASA) exposure in our in-house ALI models and EpiAirway™, along with the readouts from various analyses and assays.

#### JSC Mars-1 dust generation and characterization

The JSC Mars-1 simulant was wet ground in a planetary ball mill (Fritsch Pulverisette 6, Idar-Oberstein, Germany) for 60 min at 250 rpm using a zirconium oxide jar (25 ml). JSC Mars-1 dust (1.5 g) was suspended in 12 mL D.I. water and milled with 41 g of zirconium oxide balls (5 mm diameter). Water was chosen as dispersant to take into account the oxidative environment of the Martian atmosphere, to maximize milling efficiency and, at the same time, to minimize heat-induced mineral alterations. A zirconium oxide jar was used to avoid metal contamination. After grinding, the powder sample was sieved, and the fine fraction (<10 μm) was retained.

Mineral phase identification and morphometric and chemical characterization of the fine fraction (< 10 μm) were investigated by micro-Raman spectroscopy and automated image analysis coupled with secondary electron microscopy and energy-dispersive spectrometry (SEM‒EDS). Raman spectra were obtained with a Horiba Jobin Yvon HR800 Raman spectrometer equipped with an Olympus BX41 confocal microscope (600 grooves/mm holographic grating monochromator, high-gain Peltier-cooled CCD). A Nd solid-state laser at 532 nm was used as excitation and neutral density filters were used to avoid sample heating. Spectra were obtained on particle grains with a 100 X objective magnification, the minimum lateral resolution was ∼2 μm and the resolution along the z-axis ∼1 μm. The spectrometer was calibrated using the 520.7 cm^−1^ Raman peak of silicon before each experimental session. Spectra were collected using 10 acquisitions for 10 s in the low and high wavenumber spectral range. The SEM instrument used was a JEOL JSM IT300LV with a W source equipped with an EDS (Oxford Instruments Inca Energy 200, X-Act SDD detector), and the AZTEC Feature package software for automated image analysis. Backscattered Electron Images were acquired at 500x magnification and 15 kV accelerating voltage. Microanalysis operating conditions were 15 kV and 5 nA, 105 CPS and 30 s counting time; relative wt% errors were <1% for major elements and <5% for minor components. Standards comprise pure elements, oxides and/or silicate. Before the analysis, samples were coated with a carbon layer. More than 6000 particles were investigated.

To ensure that observed biological effects induced by JSC Mars-1 dust are not confounded by endotoxin contamination, the endotoxin level of the JSC Mars-1 dust was measured with a Pierce Chromogenic Endotoxin Quant Kit (Thermo Fisher Scientific, Rockford, IL, USA) according to the manufacturer’s instructions.

#### Exposure regimen

To determine the working concentration of exposure, we detected cell viability against different concentrations of JSC Mars-1 dust, commercial quartz Min-U-Sil 5 (positive control), and tungsten carbide (WC, negative control) ^42^ under submerged culture conditions (see supplemental information). Then, 40 (In-house PBEC ALI model) or 50 (EpiAirway^TM^ model) μl of PBS (sham) or PBS containing 3 different concentrations (1.25, 5, or 20 mg/ml, corresponding to exposure doses of 55, 222, and 890 μg/cm^2^, calculations follow) of JSC Mars-1 dust was added to the apical side of the models cultured under ALI conditions, and the cells were incubated for different durations for different experimental outcomes.

The dose of JSC Mars-1 dust (ED) was calculated as:

$$\text{Exposure}\;\text{Dose}\;\left(\text{ED}\right)=\frac{\text{Concentration}\;\left(\text{JSC}\;\text{Mars}-1\right)*\text{volume}\;\text{added}\;\text{to}\;\text{the}\;\text{model}}{\text{IS}\;\left(\text{insert}\;\text{surface}\;\text{area}\right)}$$

#### Cytotoxicity assay

The cell viability 24 hours post-exposure was determined by a lactate dehydrogenase (LDH) assay (Thermo Fisher Scientific, Rockford, IL, USA). Transepithelial electrical resistance (TEER) measurements, which can indicate cell membrane integrity, were performed before and 24 hours after simulant exposure. DNA damage was investigated by measuring 8-hydroxy-2-deoxyguanosine levels in culture medium using competitive ELISA (Invitrogen, Thermo Fisher Scientific, Rockford, IL, USA).

#### FACS

The cell models were trypsinized, washed, and resuspended in 400 μl of PBS 2 hours or 24 hours after exposure. The time points for detecting different biomarkers were selected based on our previous study.^19^^,^^43^ For reactive oxygen species (ROS) production, CellROX™ Green Reagent kit (Thermo Fisher Scientific, Rockford, IL, USA) at a concentration of 5 μM was used 2 hours post-exposure. Twenty-four hours after exposure, cellular apoptosis induction was measured with a FITC annexin V apoptosis detection kit II (BD Biosciences, San Jose, CA, USA). The surface expression of toll-like receptor (TLR)2 and TLR4 24 hours after exposure was measured by staining with antibodies against TLR2 and TLR4 (BD Biosciences, San Jose, CA, USA). Analyses were performed using a flow cytometer (LSR Fortessa™, BD Bioscience, USA) according to the manufacturer’s instructions. The flow cytometric data were analyzed using FlowJo software (BD Biosciences, San Jose, CA, USA). The data are presented as the median fluorescence intensity (MFI) for the ROS levels, TLR expression, and percentage of the apoptosis rate.

#### Cytokine multiplex assay

The basal medium from the cell models were collected 24 hours post-exposure. The secretion of cytokines, including interleukin 8 (IL-8/CXCL8) and matrix metallopeptidase 9 (MMP-9), were measured by a DouSet ELISA kit (Bio-Techne, UK). Meso Scale Discovery (MSD) measurements on the basal medium were performed according to the manufacturer’s instructions. A V-PLEX plate (MSD, Maryland, USA) was used to measure the levels of interleukin-6 (IL-6), tumor necrosis factor alpha (TNF-α), interleukin-10 (IL-10), interleukin-1beta (IL-1β), and interleukin-13 (IL-13).

#### qRT- PCR

The cell models were lysed, and mRNA were extracted with a RNeasy Micro Kit (Qiagen, Germany) 24 hours after exposure. cDNA was synthesized from 100 ng of mRNA with a cDNA synthesis kit (Applied Biosystems, Germany). cDNA (1 ng/μl) was added to a 20 μl reaction mixture. The housekeeping gene beta-actin was used as an internal control. The expression levels of caspase 3, receptor-interacting protein kinase 3 (*RIP3*), necrosis marker high mobility group box 1 (*HMGB1*), *CXCL-8*, *IL-6*, *TNF-α*, *TLR2*, *TLR4*, heme oxygenase 1 (*HMOX-1*), glutathione peroxidase 1 (*GPx1*), superoxide dismutase 3 (*SOD3*), nuclear factor kappa-light-chain-enhancer of activated B cells (*NFκB*) and nuclear factor erythroid 2-related factor 2 (*NRF2*) were calculated by the delta-delta Ct method and are presented as the fold change (2-ΔΔCt). The sequences of primers used for RT‒PCR can be found in the supplementary material (Table S1).

### Quantification and statistical analysis

The results are expressed as medians and interquartile ranges (25^th^-75^th^ percentiles). Comparisons between the sham group and different JSC Mars-1 dust exposure groups were assessed by the Friedman test followed by the Wilcoxon signed-rank test. All the data were analyzed using GraphPad Prism software (GraphPad Software, Inc. CA, US). A p value < 0.05 was considered significant.
