# Supplementary material for: Potential pulmonary toxic effects of Martian dust simulant
Source: iScience. 2025 Aug 15;28(9):113259. doi: 10.1016/j.isci.2025.113259 (PMC12496206; doi:10.1016/j.isci.2025.113259)
Supplement: Document S1. Figures S1 and S2, Table S1, and Methods S1 [file mmc1.pdf]

iScience, Volume *28*

Supplemental information

Potential pulmonary toxic effects of Martian dust simulant

Jie Ji, Jasmine R. Petriglieri, Francesco Turci, Shanzina Sompa, Per Gerde, Lars L. Karlsson, Dag Linnarsson, David J. Loftus, G. Kim Prisk, Urs Staufer, Erin M. Tranfield, Wim van Westrenen, and Lena Palmberg

Supplemental information

Supplemental figures

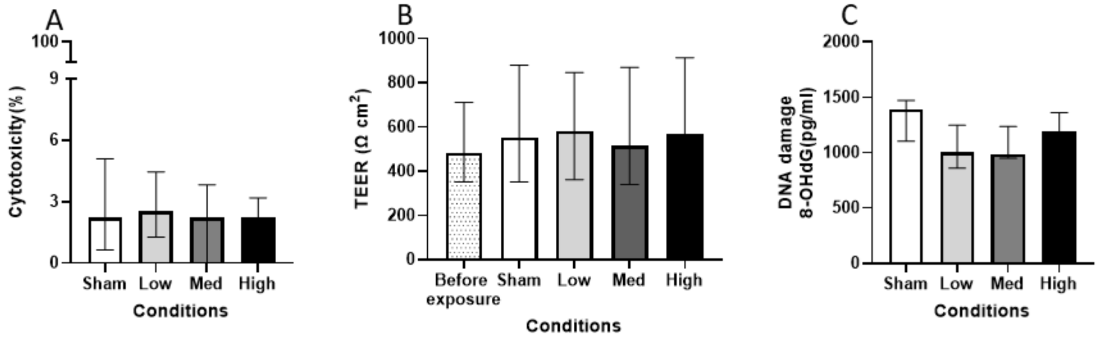

Figure S1. Cytotoxicity post JSC Mars-1 dust exposure. Cell viability (A) (N=4, n=12), Transepithelial electrical resistance (TEER) (B) (N=4, n=12), DNA damage (C) (N=4, n=4) after exposure to JSC Mars-1 dust and incubated for 24 h; 8-OHdG: 8-Hydroxy-2'-deoxyguanosine; Data presented as median and 25th -75th percentiles; % Cytotoxicity was calculated by using the formula according to the manufacturer; Med: medium.

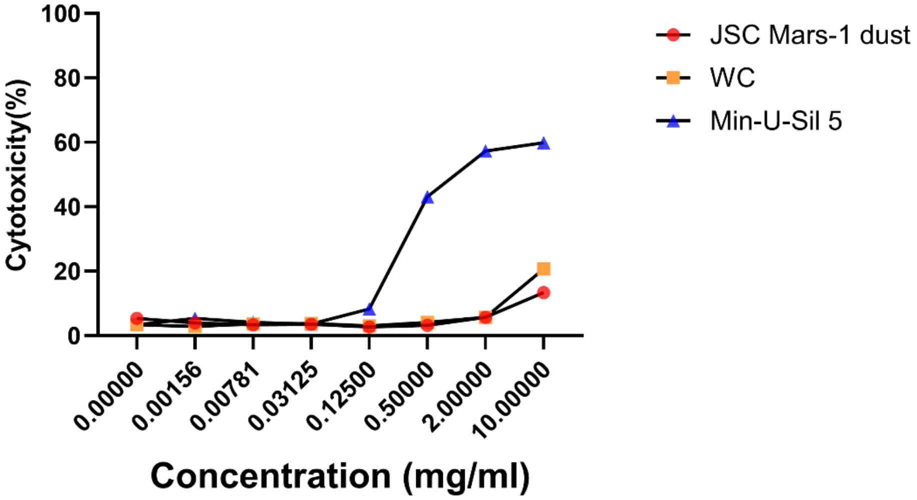

Figure S2. Cytotoxicity under submerged culture conditions. Cell viability of PBCE under submerged culture condition after exposure to different concentrations of JSC Mars-1 dust (0-10mg/ml), commercial quartz Min-U-Sil 5 (positive control), and Tungsten carbide (WC, negative control) and incubated for 24 h. Cytotoxicity (%) was calculated by using the formula according to the manufacturer.

Supplemental table

Table S1: Primers used for quantitative real-time PCR (qPCR)

Gene name	Gene Symbol	Forward Primer 5'-3'	Reverse Primer 3'-5'
Beta (β)-Actin	ACTB	CTGGGACGACATGCAGAAAA	AAGGAAGGCTGGAAGAGTGC
Caspase-3	CASP3	GGAAGCGAATCAATGGACTCTGG	GCATCGACATCTGTACCAGACC
Receptor Interacting Serine/Threonine Kinase 3	RIPK3	GCTACGATGTGGCGGTCAAGAT	TTGGTCCCAGTTCACCTTCTCG
High Mobility Group Box 1	HMGB1	GCGAAGAAACTGGGAGAGATGTG	GCATCAGGCTTTCCTTTAGCTCG
Heme oxygenase 1	HMOX1	TTCAAGCAGCTCTACCGCTC	GGGGGCAGAATCTTGCACTTT
Glutathione Peroxidase1	GPX1	GTGCTCGGCTTCCCGTGCAAC	CTCGAAGAGCATGAAGTTGGGC
Superoxide Dismutase 3	SOD3	ACGCTGGCGAGGACGACCTG	GCTTCTTGCGCTCTGAGTGCTC
C-X-C Motif Chemokine Ligand 8	CXCL8	GCTCTGTGTGAAGGTGCAGTT	GGCACAGTGGAACAAGGACT
Interleukin 6	IL6	ACCCCCAGGAGAAGATTCCA	CACCAGGCAAGTCTCCTCATT
Tumor necrosis factor alpha	TNFα	AGCCCATGTTGTAGCAAACC	ACATTGGGTCCCCCAGGATA
Toll-like receptor 2	TLR2	CTCATTGTGCCCATTGCTCTT	TCCAGTGCTTCAACCCACAAC
Toll-like receptor 4	TLR4	GGCCATTGCTGCCAACAT	CAACAATCACCTTTCGGCTTTT
Nuclear factor kappa-light-chain-enhancer of activated B cells	NFKB	AAGAGGAGGTTTCGCCACCG	TTGCAGATTTTGACCTGAGGGT
Nuclear factor erythroid 2-related factor 2	NRF2	CACATCCAGTCAGAAACCAGTGG	GGAATGTCTGCGCCAAAAGCTG

Methods S1: Exposure dose determination

The PBEC were cultured submerged in a 24-well plate with PneumaCult™-Ex expand medium for 48 hours. Then, 850 µl of fresh medium with serial dilutions of particles (0 mg/ml to 10 mg/ml) was added, and the mixture was incubated for 24 hours. The medium was collected for lactate dehydrogenase (LDH) assay (Thermo Fisher Scientific Rockford, IL, USA). To ensure the greatest possibility of conversion from submerge culture to ALI culture, the metric of particles per surface area was chosen for the calculation. Based on our calculation, $\frac{\mathrm{C_{ALI}} \times 40\mu\mathrm{l}}{0.9\mathrm{cm}^2} = \frac{\mathrm{C_{submerge}} \times 850\mu\mathrm{l}}{1.9\mathrm{cm}^2}$ (0.9 cm^2 for the insert surface area and 1.9 cm^2 for the plate well surface area); thus, $C_{ALI} = 10 \times C_{submerge}$. Figure S2 shows that under submerged culture conditions, the cytotoxicity of PBEC increased dramatically in the presence of the positive control particles above a concentration of 0.125 mg/ml; however, for JSC Mars-1 and the negative control particles, there were no cytotoxic effect even at a concentration of 2 mg/ml. Quartz is widely recognized as a benchmark toxic particle due to its well-documented ability to induce severe pulmonary inflammation and fibrosis following exposure 1,2 . The observed difference in cytotoxicity between quartz and JSC Mars-1 in our study indicates that, at the tested exposure levels (0.125, 0.5, and 2 mg/ml), Martian dust simulant may present a lower acute cytotoxic risk. However, this does not exclude the possibility of long-term adverse effects, such as persistent inflammation or fibrotic remodeling, which warrant further investigation. To ensure a relevant assessment in ALI models, we exposed our models to 1.25, 5, and 20 mg/ml of JSC Mars-1 dust, corresponding to 10 times the exposure levels used in submerged conditions. This resulted in surface doses of 55, 222, and 890 µg/cm^2 for the low-, medium-, and high-exposure groups, respectively.

Supplemental references

1. Hnizdo, E., and Vallyathan, V. (2003). Chronic obstructive pulmonary disease due to occupational exposure to silica dust: a review of epidemiological and pathological evidence. Occup Environ Med *60*, 237-243. 10.1136/oem.60.4.237.
2. Reiser, K.M., and Last, J.A. (1979). Silicosis and fibrogenesis: fact and artifact. Toxicology *13*, 51-72.
